# 

**Published:** 1887-02-19

**Authors:** 


### CONTENTS.

**Unite and Co-operate** - 343

**Words of Consolation.**—XXI. Nurses' Trials—Martha and Mary - 344

**A Noble Profession: Nursing the Sick.**—Nursing Institutions and Metropolitan Hospitals. By Miss E. G. Manson, Matron of St. Bartholomew's Hospital - 345

**Employment for Epileptics.** By Miss Nina Paget - 347

**The Mildmay Mission Hospital.** By A Young Hand - 349

**Notes and News.**—Miscellaneous Items.—The Payment of Nurses.—Lectures for Nurses.—The Frome Cottage Hospital.—Derbyshire Hospital for Sick Children.—Vacancies.—Queen's Hospital, Birmingham.—Cremation.—Pianos in Hospitals, etc., etc. - 350

**Annotations.**—Waking up at the Dorset County Hospital.—A Third Hospital for Glasgow.—Nurses at Play - 353

**Deaf-mutes and Consanguineous Marriages** 353

**Infectious Fevers and Diseases in London.** By "Public Health" - 354

**Digestion and Dyspepsia** - 354

**The Quilter Prizes.**—Hospital Housekeeping and Management - 355

**Poetry.**—Growing Old. By A Cottage Hospital Nurse 356

**Till the Doctor Comes.**—XI. Fomentations, Lotions, and Bed-sores - 356

**A Great Opportunity for Good** - 357

**The Children's Corner.**—Puzzles. Answers, etc. - 357

**Amusements with Profit** - 358

**In- and Out-Patients' Hospital Diary** - 359

